# A Case of Uncommon Acuteness of the Sense of Vision

**Published:** 1846-12

**Authors:** 


6. *A Case of Uncommon Acuteness of the Sense of Vision.*—There is living in this region a young man of 23 or 24 years of age, who is reported as being able to see, with his natural eye, *animalculæ* in common well and spring water. This faculty was noticed when he was some 15 or 16 years of age, by persons for whom he was at work, in consequence of his refusing very often to drink water handed to him, in which nothing could be discovered by common eyes. I made some experiments with him, enough to be satisfied that his case was no hoax; and did intend to have made more, but have lost sight of him, and suppose he has left the neighborhood. His complexion is fair; temperament sanguine; eyes blue, less than the common size, with very small pupils.—*Ibid.*

